# Partial Purification and Characterization of the Recombinant Benzaldehyde Dehydrogenase from *Rhodococcus ruber* UKMP-5M

**DOI:** 10.15171/ijb.1344

**Published:** 2017-03

**Authors:** Arezoo Tavakoli, Ainon Hamzah

**Affiliations:** ^1^ Department of Nursing, Faculty of Nursing, Islamic Azad University, Eghlid, P.O.Box: 73815-114, Iran; ^2^School of Biosciences and Biotechnology, Faculty Science and Technology, National University of Malaysia, Selangor, P.O.Box:43600, Malaysia

**Keywords:** Benzaldehyde dehydrogenase, Purification, *Rhodococcus ruber* UKMP-5M

## Abstract

**Background:**

Benzaldehyde dehydrogenase (BZDH) is encoded by the *xylC* that catalyzes the conversion of benzaldehyde into benzoate in many pathways such as toluene degradation.

**Objectives:**

In this study, the *xylC* gene from *Rhodococcus ruber* UKMP-5M was expressed in *Escherichia coli*, purified, and characterized.

**Materials and Methods:**

The *xylC* was amplified and cloned in *E. coli*. The recombinant plasmid *pGEMT-xylC* was digested by NdeI and HindIII to construct plasmid *pET28b-xylC* and transformed in *E. coli* BL21 (DE3). Expression of the recombinant protein was induced by 1 mM isopropyl β-D-thiogalactoside (IPTG) at 37°C. The BZDH was purified by ion exchange chromatography, in which the product was an NAD-dependent enzyme using benzaldehyde as a substrate for enzyme characterization. The end metabolite was identified via gas chromatography mass spectrometry (GC-MS).

**Results:**

The recombinant BZDH is 27 kDa, purified by ion exchange chromatography. The activity of BZDH was 9.4 U.μL^-1^ The optimum pH and temperature were 8.5 and 25ºC, respectively. The Michaelis constant (K_m_) and maximum velocity (V_max_) were 4.2 mM and 19.7 U.mL^-1^, respectively. The metabolite of BZDH was benzene carboxylic acid as determined by GC-MS analysis.

**Conclusions:**

BZDH has the ability to degrade benzaldehyde to less toxic compounds. The BZDH is a critical enzyme for the degradation of aromatic hydrocarbons in *Rhodococcus* sp. The BZDH from R. ruber UKMP-5M is showed similar function with other aldehyde dehydrogenases.

## 1. Background

*Rhodococcus ruber* UKMP-5M is a hydrocarbon degrading bacteria through catabolic pathway crude oil and toluene (1). The toluene degradation pathway consists of two steps. The first is an upper pathway induced by toluene, which catalyzes the conversion of aromatic hydrocarbons to their carboxylic acid derivatives. The second is the lower pathway induced by benzoic acid (the alternative pathway); the product that is supplied by the upper pathway (2). Benzaldehyde dehydrogenase (BZDH) is an important enzyme involved in the upper pathway of toluene and xylene degradation. This enzyme is a member of aldehyde dehydrogenases, which detoxifies benzaldehyde to carboxylic acid compounds via irreversible oxidation reaction (3). Two types of BZDH are determined: type I induced by benzoylformate and involved in the mandelate pathway (4) and the type II induced by benzaldehyde involved in toluene and xylene degradation pathway (5). Type II of BZDH has been reported by many bacteria such as *Pseudomonas putida* (2),* Acientobacter calcoaceticus* (4),* Rhodococcus rhodochrous* OFS (6) and* Pseudoxanthomonas spadix* (7). The BZDH in *P. putida* mt-2 is encoded by the TOL plasmid (pWW0) to catalyze various mono aromatic alcohols and aldehydes (8). More catalytic efficiency with broad substrate specificity for BZDH has been shown in *A. calcoaceticus* (3,8). This paper describes the expression, purification, and characterization of BZDH from *R. ruber* UKMP-5M.

## 2. Objectives

Purification and characterization of BZDH from *Rhodococcus ruber* UKMP-5M was studied.

## 3. Materials and Methods

### 3.1. Cloning of the xylC Gene for BZDH

The total DNA of bacteria was extracted using Wizard genomic DNA-purification kit (Promega, Madison, USA). The *xyl*C gene was amplified in an automated thermal cycler (Bio-Rad, California USA) using specific primers designed based on genome sequences from *R. ruber* UKMP-5M. Restriction enzyme recognition sites were underlined in the sequences. The forward oligonucleotide containing an *Nde*I site (CAꞌTATG) and reverse oligonucleotide with a *Hind*III site (AꞌAGCTT).

Forward (xylC): 5'CATATGATGTCTCCTTCACCGGTTCCACCCCGG 3'

Reverse (xylC): 5'AAGCTTTCAGAAGGGGTAACCGGGCACGTCGC 3'

The purified DNA (~0.8 kb) was ligated into pGEM^®^-T Easy vector (Promega, Madison, USA) and transformed into competent cells of *E. coli* DH5α using a heat shock method at 42ºC for 50 s. The transformed *E. coli* was cultured on LB agar containing ampicillin (Sigma, Saint Louis USA) (50 µg.mL^-1^), 50 mg.mL^-1^ 5-bromo-4-chloro-3-indoyl-b-D*-*galactopyranoside (X-Gal) (Promega, Madison, USA) and 100 mM isopropyl b-D*-*1-thiogalacto pyranoside (IPTG) (Sigma -Aldrich, Taufkirchen, Germany) for 16 h. The positive transformants were screened from white colonies by PCR. The plasmid *pGEMT-xylC* was extracted via QIAprep Miniprep kit (Qiagen, Hilden, Germany) according to manufacturer’s instructions and analyzed using 1% agarose gel. Subsequently, the size and accuracy of plasmid *pGEMT-xylC* was determined by a supercoiled ladder (Promega, Madison, USA) as standard and PCR. The *xyl*C fragments from *pGEMT-xylC* and *pET 28b* (Novagen, Madison, USA) were excised using the *Nde*I and *Hind*III restriction enzymes and recovered from agarose gel. The purified *xylC* was inserted into linearized *pET 28b* using T4 DNA ligase for 16 h at 16°C. The cloned was transformed into *E. coli* DH5α using heat shock method and the transformed cells were cultured onto LB agar containing kanamycin (50 mg.mL^-1^). The recombinant plasmid *pET28b-xylC* was extracted from positive transformants and screened by PCR‏. The nucleotide sequences of the plasmids *pGEMT-xylC* and *pET28b-xylC* were determined by DNA sequencing using xylC, M13 and T7 primers (universal primers). The sequencing data were analyzed by VecScreen, BLASTP, and BLASTN (9).

### 3.2. Expression of BZDH

The plasmid *pET28b-xylC* was transformed into *E. coli* BL21 (DE3). A pre-culture from the transformant was prepared in LB broth and incubated at 37ºC to reach optical density OD_550_ ~ 0.5. The standard inoculums (10%) were diluted to minimal salt medium (MSM) (10) induced by 0.5-2 mM benzaldehyde. The culture was incubated at 30°C, 150 rpm for 3 days and OD_550_ was measured. The control was run in parallel condition with *E*. *coli* BL21 (DE3) without recombinant plasmid. The pre-culture was prepared by inoculating a single colony of *E. coli* BL21 (DE3) into 10 mL LB broth containing 50 mg.mL^-1^ kanamycin and shaken at 37°C, 250 rpm for 16 h. The cells were centrifuged at 4ºC, 4000 rpm for 15 min and the supernatant was discarded. The resuspended pellet was diluted 5-fold (50 mL) and incubated to adjust an OD_550_ 0.6. Culture (1 mL) was collected as an uninduced sample (control) and the culture was induced by adding IPTG (0.01-1 mM) at 37°C after 1, 2, 4, 6 and 16 h of incubation. The harvested cells were dissolved in lysis buffer (50 mM NaH_2_PO_4_ with 300 mM NaCl) (pH 8.0) and 1 μg.mL^-1^ lysozyme and 0.1 mM phenylmethyl sulfonyl fluoride (PMSF) (Sigma-Aldrich, Taufkirchen, Germany) added to the mixture and incubated in ice for 30 min. The cells were disrupted by sonicator (Sonics-vibra cell, Ontario, Canada) at 20 s pulses with 5 min rest for 30 min. The crude lysate was centrifuged at 12000* ×g* for 60 min at 4°C. The supernatant and the pellet were separated and 15 mL of each sample loaded into 12% sodium dodecyl sulphate-polyacrylamide gel and run at 150 V. Expression was confirmed through western blot when the protein was transferred from the gel onto a nitrocellulose membrane at a constant voltage of 15 V for 45 min using Trans-Blot SD semi-dry electrophoretic transfer cell (Bio-rad, California USA). The target protein was determined after reaction with a monoclonal antibody.

### 3.3. Purification of BZDH

The BZDH was overexpressed in a 3L batch in optimal condition. The inclusion bodies were formed after high expression of target protein. Thus, some treatments such as use of lysozyme and sonication were applied to reduce the viscosity of the suspension followed by centrifugation at 12000 *×g* for 60 min at 4ºC. The supernatant (25 mL) contained the desired protein and filtered with 0.22 mm membrane filter before start of purification. Purification was carried out by AKTA prime (No 1314455 Sweden; GE Healthcare, Uppsala, Sweden) using ion exchange chromatography according to the manuals’ instructions. In the first step, machine and column were washed stepwise with buffer A (wash buffer) containing 20 mM Tris or bis-tris (pH 6-8) with flow rate of 1 mL.min^-1^ and filtered sample was injected into the Hi Trap DEAE column (i.d × 0.7 × 2.5 cm) with flow rate of 0.8 mL.min^-1^ The enzyme was eluted with a linear gradient of NaCl (1 M) in buffer B (elution buffer) containing buffer A with 1 M NaCl. The machine and column were re-equilibrated with buffer A in the last step. The collected fractions from bound and unbound proteins were analyzed using SDS-PAGE and western blot. The protein was concentrated by a vivaspin or Amicon (Millipore, Hannover, Germany) protein column to maximum volume of 3 mL and the concentration of purified BZDH was measured using bicinchoninic acid (BCA) method at OD_562_ (11). Protein degradation during purification was reduced by adding dithiothreitol (DTT) or 2-mercaptoethanol (2-ME) into buffers at a low concentration of 0.5 mM.

### 3.4. Characterization of Purified BZDH

In BZDH reaction, nicotineamide adenine dinucleotide (β-NAD) was converted to a reduced form of NADH. All assay measurements were performed in triplicate.

#### 3.4.1. Enzyme Assay

The mixture was prepared as follows: buffer (50 mM glycin-NaOH) 20 mL; H_2_O 119.15 ml and substrate (benzaldehyde) at final concentration of 0.85 mM; b-NAD (5 mM) 40 mL. The reaction was started after that 20 mL of purified BZDH was added and the reaction was monitored for 10 min with 1 min intervals at 25°C at 340 nm. The activity of enzyme was calculated by general equation based on unit.mL^-1^.

One-unit enzyme activity is the amount of enzyme that catalyzes the conversion of 1.0 mM of substrate to the expected product per min at a standard assay condition (12).

#### 3.4.2. Determination of Optimal pH and Temperature

The optimum pH was prepared in the standard assay conditions described before, except the following buffer systems from 6-11 were used: 50 mM K_2_HPO_4_ (pH 6-8), 50 mM Tris buffer (pH 8-10), and 50 mM NaHCO_3_ (pH 10-11) at 0.5 intervals. The mixture was incubated for 3 min at 25ºC and OD_340_ was measured.

At optimal pH, the temperature was adjusted to 4, 20, 25, 37, 40, 50, 60, 70 and 80°C and enzyme assays were carried out. The reaction was stopped after 3 min and the absorbance was measured at OD_340_ .

#### 3.4.3. The Kinetic Study of the Enzyme

The effect of substrate concentration on enzyme activity was evaluated by maximum velocity (V_max_) and Michaelis constant (K_m_) by varying concentration of benzaldehyde at the range of 0.005-4 mM to a total adjusted volume of 200 mL. The mixture incubated for 3 min at 25°C and OD_340_ was measured. K_m_ and V_max_ were calculated from Lineweaver-Burk plots.

#### 3.4.4. Gas Chromatography-Mass Spectrometry (GC-MS) Analysis

The mixture was pre-incubated for 5 min and reaction was stopped by adding 300 mL of 0.1 M HCl. The protein was separated by Vivspin 500 (Sartorius, Gottingen, Germany) and 300 mL diethyl ether was added to residual liquid. The upper layer (1 mL) of volatile phase was injected into the injection port of the GC device.

## 4. Results

**Figure 1:**
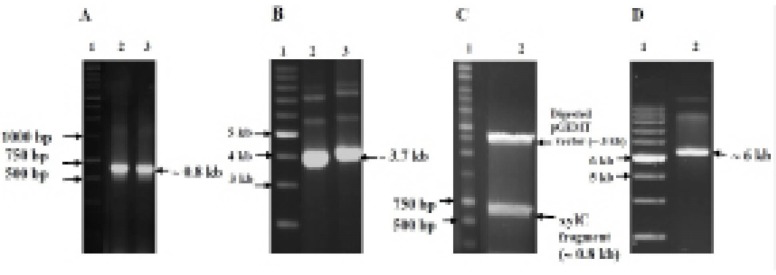
Agarose gel electrophoresis analysis of xylC. (A) DNA amplifi cation; Lane 1: 1 kb DNA Ladder, Lane 2, 3: PCR product before and after DNA purification, (B) Analysis of *pGEMT-xylC*. Lane 1: DNA Super-coiled ladder, Lane 2-3: Extracted *pGEMT-xylC*, (C) Double digestion of *pGEMT-xylC*. Lane 1: 1 kb DNA Ladder, Lane 2: DNA fragment xylC after being cleaved with NdeI and *Hind*III, (D) Analysis of pET *28b-xylC*; Lane 1: DNA Supercoiled ladder, Lane 2: Extracted pET *28b-xylC*.

*Rhodococcus ruber* UKMP-5M was isolated from oil-contaminated soils in Malaysia. The *xyl*C gene was amplified at 64°C and the resulting product was ~ 0.8 kb (Fig. 1A). The recombinant plasmid pGEMT-xylC was constructed, successfully transformed into *E. coli* DH5α and extracted from the positive transformants (Fig. 1B). The inserted fragment xylC was excised with double digestion using *Nde*I and *Hind*III (Fig. 1C). The xylC fragment was ligated into pET 28b at 15ºC to form the recombinant plasmid *pET 28b-xylC*, which was 6.2 kb, consisting of pET 28b (5.4 kb) and xylC (792 bp) (Fig. 1D). The highest growth of transformant *E. coli* BL21 containing *pET 28b-xylC* was determined when the cells incubated in 0.5 mM benzaldehyde for 24 h.

**Figure 2:**
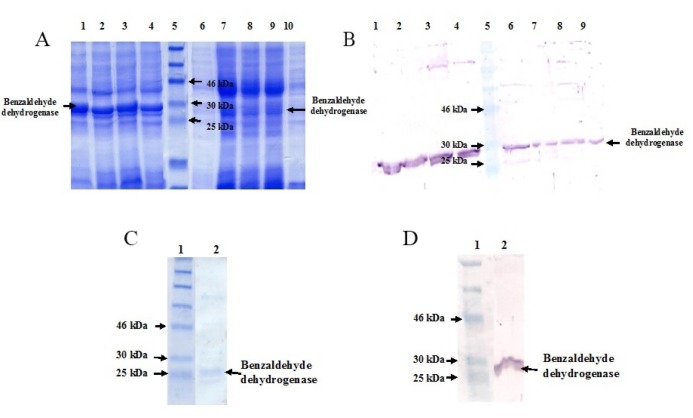
Analysis of BZDH before and after purification. (A) SDS-PAGE after BZDH expression: Lanes 1-4: Cell free soluble extract (Supernatants) at 2, 4, 6, 16 h, Lane 5: Molecular weight marker (7-175 kDa), Lane 6-9: Insoluble extract induced at 2, 4, 6 and 16 h. The small amount of BZDH was determined in pellet because of inclusion bodies, Lane 10: Uninduced sample as negative controls. (B) Western blot after BZDH expression: Lanes 1-4: Cell free soluble extract (Supernatants) at 2, 4, 6 and 16 h, Lane 5: Molecular weight marker (7-175 kDa), Lanes 6-9: Insoluble extract (pellets) at 2, 4, 6 and 16 h. (C) SDS-PAGE after BZDH purification; Lanes 1: Molecular weight marker (7-175 kDa), Lanes 2: purified benzaldehyde dehydrogenase. (D)Western blot after BZDH purification: Lane 1: Molecular mass marker, Lane 2: purified benzaldehyde dehydrogenase.

The transformed *E. coli* BL21 (DE3) was induced with 1 mM IPTG at 37ºC, which BZDH was successfully expressed for 2, 4, 6, and 16 h (Figs. 2A, B). The expression reached its highest level at 4 h post induction. A residual amount of BZDH was shown in pellet after SDS-PAGE, because of inclusion bodies formation. Using higher concentration of lysozyme in lysis buffer, high speed and long-time centrifugation reduced the protein aggregation. A BZDH protein has an approximate molecular weight of 27 kDa. The purified BZDH showed a single band on SDS-PAGE and the result was confirmed by western blot (Figs. 2C, D). The total concentration of BZDH was 1.18 mg.mL^-1^ as determined by BCA method. The results of BZDH purification from *R. ruber* UKMP-5M by anion exchange chromatography is summarized in Table 1, showing that the BZDH protein was purified at 14 folds with 85% yield.

**Table 1:** Summary of BZDH purification method from *R. ruber* UKMP-5M.

**Purification stage**	**Volume** **(mL)**	**Total activity** **(Unit)**	**Total protein (mg)**	**Specific activity (U.mg** ^-1^ **)**	**Yields (%)**	**Purification folds**
Cell-free extract	30	454	189	2.4	100	1
Ion exchange chromatography	10	389.4	11.8	33	85	14

**Figure 3:**
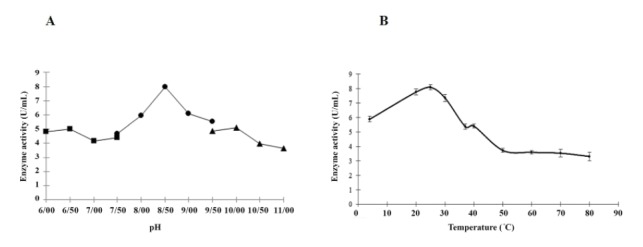
effect of physical parameters on BZDH activity; (A) effect of diff erent pH on BZDH activity. The following buff ers were used: (■) 50 mM K_2_HPO_4_ (pH 6-8), (●) 50 mM Tris-buff er (pH 8-10), (▲) 50 mM NaHCO_3_ (pH 10 -11). The incubation time was 3 min.(B) effect of diff erent temperature on BZDH activity. Enzyme was assayed using 50 mM Tris-buff er at pH 8.5. The incubation time was 3 min.

Optimum BZDH activity was at 9 min with the highest activity of 9.4 U.mL^-1^. The effect of different pHs on BZDH activity exhibited the highest level at pH 8.5 (Fig. 3A). The optimum temperature for BZDH was 25°C (Fig. 3B). Enzymatic activity decreased when the incubation temperature reached 50ºC (50% reduction in maximum activity). V_max_ for the BZDH was 19.72 U.mL^-1^ and K_m_ was 4.2 mM. The BZDH utilized benzaldehyde and the product was benzene carboxylic acid at a retention time of 12.5 min as determined by GC-MS.

## 5. Discussion

The BZDH encoded by *xyl*C was involved in the bioconversion of benzaldehyde to benzene compounds (4)‏. The sequence analysis showed the similarity of *xyl*C from *R. ruber* UKMP-5M with *R. aetherivorans* I24 (99%), NAD-dependent aldehyde dehydrogenase *Saccharomonospora cyanea* NA-134 (91%) and NAD-aldehyde dehydrogenase *Rhodococcus* sp. P14 (91%). However, the *xyl*C from *R. ruber* UKMP-5M (264 amino acids) was shorter than many other *xyl*C genes in other bacteria such *A. calcoaceticus* (484 aa) (13) and *R. erythroplis* (454 aa) (14). As a result, the molecular weight of BZDH in *R. ruber* UKMP-5M (27 kDa) that was less than similar mass in *Pseudomonas* sp strain DJ77 55 kDa (15), *P. putida* CSV86 (14) and *P. putida* sp (16) were 57 kDa. The BZDH sequence from *R. ruber* UKMP-5M was homologous with half of terminal part (C-terminal) of the other aldehyde dehydrogenases. However, another aldehyde dehydrogenase (Sequence ID spQ29491.1 ALDH2) was estimated at about 240 amino acids. The enzyme activity of BZDH was 9.4 U.mL^-1^ nearby to BZDH activity from *P. putida* MT53 (9.7 U.mL^-1^) (17). The optimal pH for the activity of BZDH from *R. ruber* UKMP-5M was 8.5, close to other studies by *Pseudomonas fluorescens* strain A.3.12 and *Pseudomonas stutzeri* ST-201 was 8.5 (18), *Antirrhinum majus* 8.0, (5) *P. putida* 9.0 (19), 9.3 for *A. baylyi* (20) and *P. putida* MT53 (16), 9.5 for A.* calcoaceticus* (21) and 9.6 for *P. putida* CSV86 (2). The stringent pH requirement for BZDH activity at the range of 8.0-10 suggests that the BZDH is highly specific with respect to hydrogen ion concentration (16,19).

The optimum temperature for BZDH activity from *R. ruber* UKMP-5M and *P. stutzeri* ST-201 (18) was 25°C. The activity of BZDH was not stable for a long period and BZDH from *R. ruber* UKMP-5M, *P. putida* pWW0 MT53 and *A. calcoaceticus* (17) were losing 50% of enzyme activity at 50ºC within a period of one to 5 min.

The K_m_ value of BZDH from *R. ruber* UKMP-5M (4.2 mM) was much higher to what was reported earlier. The K_m_ value for BZDH was 460 µM for *P. putida* (16), 1.4 µM for *P. putida* CSV86 (2), 2.5 mM for *P. putida* pWW0 (19), 0.63 mM for *A. calcoaceticus*, 0.79 mM for *P. putida* (17) and 7 mM reported for *P. stutzeri* ST-201 (18), indicate a different variation in specificity for BZDH, even within the same genus. The high K_m_ value for BZDH from* R. ruber* UKMP-5M could be explained by a limited number of active sites when compared with other aldehyde dehydrogenases. As a result, the enzyme showed low affinity for benzaldehyde as a substrate, which requires to have greater concentration of substrate to achieve V_max_ and the enzyme activity was highly dependent on substrate. It is possible that BZDH from *R. ruber* UKMP-5M has a preference for other substituted of benzaldehyde than benzaldehyde, which also showed in other BZDHs (21). The BZDH from *R. ruber* UKMP-5M showed lower V_max_ compared to similar BZDH in *P. putida* (104 U.mL^-1^), *A*. *calcoaceticus* (63.5 U.mL^-1^) (17) and 48 U.mL^-1^ for *P. putida* (19), which lead to the low rate of catalysis. The high K_m_ value and low V_max_ for BZDH from *R. ruber* UKMP-5M suggest that this enzyme may be active in high concentration of benzaldehyde, although it is slow in catalytic reaction and may be applicable for biodegradation in high contaminated area with hydrocarbons. The BZDH from *R. ruber* UKMP-5M is able to convert benzaldehyde to benzene derivatives. The products of BZDH in *P. putida* CSV86 was benzoic acid (22), benzoate and its derivatives (16,2) as determined by GC-MS.

The achievements of this paper show that benzaldehyde dehydrogenase is a NAD-dependent enzyme, important for hydrocarbon degradation through *R. ruber* UKMP-5M. The enzyme revealed similar characteristics to other aldehyde dehydrogenase even though it has smaller mass than others. However, it is apparent that benzaldehyde dehydrogenase has a catalytic mechanism differing from classical mechanisms, resulting in low affinity and slow catalysis for benzaldehyde. In spite of this fact, it could be possible that the other enzymes of *R. ruber* UKMP-5M interfere in hydrocarbon biodegradation. The previous studies indicated that although aldehyde dehydrogenases are similar to each other in terms of many properties, they are different with respect to features such as cofactor, substrate specifities, or genetic regulation. The results presented in this paper provide a starting point for a detailed molecular comparison of isolated BZDH *R. ruber* UKMP-5M with other BZDH.

## Acknowledgments

The work was supported by grant number STGL-003-2007 and 02-01-02-SF0408.
